# *Malasseziafurfur* bloodstream infection: still a diagnostic challenge in clinical practice

**DOI:** 10.1016/j.mmcr.2024.100657

**Published:** 2024-07-03

**Authors:** Rosalba Petruccelli, Terenzio Cosio, Valeria Camicia, Carlotta Fiorilla, Roberta Gaziano, Cartesio D'Agostini

**Affiliations:** a Laboratory of Clinical Microbiology, Policlinico Tor Vergata, 00133, Rome, Italy; b Department of Experimental Medicine, University of Rome “Tor Vergata”, 00133, Rome, Italy; c Dynamyc Research Team 7380, Université de Paris-Est-Créteil, F-94000, Créteil, France; d Department of Biochemical Sciences, Catholic University “Our Lady of Good Counsel”, 1000, Tirana, Albania

**Keywords:** *Malassezia furfur*, Fungemia, Fast microbiology, Acute lymphoblastic leukemia, Unmet need

## Abstract

The opportunistic fungus *Malassezia furfur* (*M. furfur*) can cause either cutaneous or systemic infections. We report a case of *M. furfur* fungemia in a 22-year-old male with T-cell Acute Lymphoblastic Leukemia (T-ALL) who developed concomitant *Bacillus cereus* (*B. cereus*) septicemia. The fungal infection was diagnosed by microscopic examination and culture-based methods, while automated blood culture systems and molecular approaches failed in identifying the fungus. Despite appropriate therapy, the patient died 18 days after the hospitalization.

## 1 Introduction

*Malassezia furfur* is a lipophilic, yeast-like fungus that normally colonizes the human skin as commensal organism. This fungus mainly causes a wide spectrum of cutaneous diseases, although in recent years the number of reports of invasive infections caused by this fungal pathogen is constantly increasing [1]. *M. furfur* is the most encountered species from bloodstream infections within the *Malassezia* genus, followed by *M. pachydermatis* and *M. sympodialis*, especially in premature neonates and immunocompromised hosts [[1], [2], [3]]. Intravenous injections via a central venous catheter (CVC) represents the major risk factor for systemic *Malassezia* infections in both adults and neonates, followed by total parenteral nutrition (TPN) and invasive procedures [4,5].

The absence of specific clinical manifestations during deeply invasive infections and the difficulty to identify the fungus by routine clinical blood cultures, may often hinder the diagnosis of *M. furfur* fungemia. In this view, a rapid diagnosis is still a challenge in clinical microbiology, leading to the introduction of the fast microbiology, synonym of successful automation [6].

Time reduction in identification and antimicrobial susceptibility tests can lead to a better and adequate therapeutic approach, especially when multi-drug resistant (MDR) strains or life-threatening infections, such as invasive fungal diseases (IFDs), request a prompt diagnosis [6]. Despite the growing of new tools in the fast microbiology, there are still unmet needs for rapid diagnostic testing in clinical microbiology, especially when we face out systemic fungal infections, due to the high mortality rate particularly in immunocompromised patient [6].

Here, we report a case of systemic infection due to *Malassezia furfur* masked by *Bacillus cereus* septicemia that was not detected by automated blood culture systems and molecular methods.

## 2 Case presentation

A 22-year-old Caucasian male affected by T-ALL (deletion of the long arm of chromosome 6), in treatment with Navitoclax plus Venetoclax-based savage therapy from the last months (Day −30) was admitted to the Emergency Department of our tertiary care University hospital (Day 0) with pleuritic chest pain, dyspnea, and fever that started 3 days prior to admission. He was hospitalized a week earlier at a different medical center for routine treatment of T-ALL through CVC (Groshong line) (Day −7). No recent contact with domestic animals or new drug intake were reported. At the admission in our center (Day 0), the patient presented a weight of 55 Kg and height of 180 cm. Physical examination of the patient revealed a temperature of 37.9 °C, blood pressure 121/76 mmHg, pulse 87 beats/min, and respiratory rate of 16 breaths/min with an oxygen saturation of 98 %. Blood tests revealed Hb 7.6 g/dL, Platelets 7.000/mmc, White Blood Cells 1.580/mmc, Neutrophils 720/mmc. High resolution chest computer tomography (CT) showed a pleural effusion layer on the left with a maximum thickness of 23 mm at the basal site, with a concomitant minimal amount of contiguous atelectatic parenchyma. Limited “ground glass” areas of altered parenchymal density in the right middle lobe, suggesting for T-ALL progression. The main airways are patent. He was admitted to the hematological ward for pleural drainage, receiving TPN and follow-up monitoring.

**Fig. 1:**
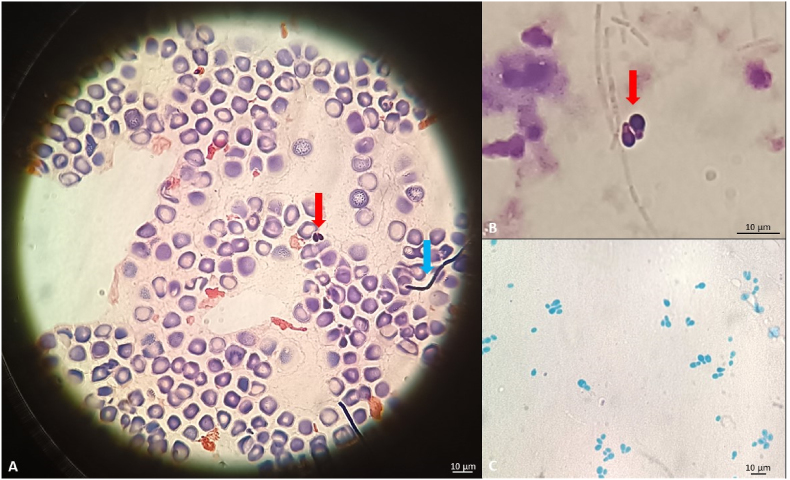
Gram stain smear from blood samples at Day 0 shows the presence of Gram-positive small cells, elliptical in shape (red arrow) and Gram-positive slender bacilli with rounded ends, arranged singly in short chains (blue arrow) (A); Gram-positive yeast-like cells from blood smear (B). Lactophenol blue wet mount of a portion of the colony on CAN2 (Day 14) shows spherical to elliptic yeast-like cells and short pseudohyphal elements with several monopolar budding cells (C). All images are at 100x magnification.

Due to the clinical suspicion of a systemic infection in course of neutropenic fever, three sets of peripheral blood cultures (Day 0) and three from the CVC were collected to detect microbial pathogens by using the BacT/Alert Virtuo® automated system (VIRTUO™, bioMérieux Inc., Hazelwood, MO). Aerobic blood culture from CVC were positive 4.3 hours after incubation. In parallel, blood smear microscopic examination of collected cultures from CVC and peripheral blood revealed the presence of Gram-positive bacilli and rare yeast-like cells (Fig. 1A and B). The ePlex® system, a double molecular diagnostic test, was used for a rapid identification of bacterial and fungal species. While the Gram-positive panel (ePlex® BCID-GP) revealed the presence of *B. cereus*, no fungal pathogens were detected by the ePlex® BCID Fungal Pathogens Panel. Given the suspicion of bloodstream co-infections by bacteria and fungi, we carried out sub-cultures on Sabouraud dextrose agar (SDA) and on Agar chromID™ Candida (CAN2) at 25 and 37 °C from positive blood samples. The patient was treated intravenously (IV) with piperacillin/tazobactam at a dose of 4,5 g q6h and fluconazole 400 mg/die. On the same day, urine cultures for bacterial and fungal infections were negative nor serum galactomannan and procalcitonin measurements. The (1,3)-β-D-glucan measurement in blood resulted indeterminate.

On Day +3, there was a reduction in hyperthermia and clinical improvement of the patient despite the presence of thoracic and lumbar pain. Pleural drainage cytological analyses showed Atypical T lymphocytic infiltrate, confirming the presence of the neoplasm as the causative agent of the pleural effusion.

On Day +4, considering the clinical improvement but the lumbar pain, magnetic resonance imaging showed minimal edematous imbibition of the paravertebral muscles in the lumbar region. As a lateral finding, the presence of left pleural effusion (6mm) was reported. On Day +7 there was a relapse of the T-ALL due to the reappearance of a circulating blast rate equal to approximately 50 %. On Day +9, vincristine 2 mg was administered for cytoreductive purposes in association with steroid plus Navitoclax and Venetoclax.

After 11 days (Day +11), on CAN2 plate incubated at room temperature, few large white-pale pink and wrinkled colonies developed but direct Matrix Assisted Laser Desorption/Ionization-Time of Flight mass spectrometry (MALDI-TOF MS) Biotyper® (Bruker Daltonics, Bremen, Germany) identification failed in identifying the microorganism, suggesting a *Malassezia furfur* with low score (1.20). According to the suggestion, a subculture on SDA supplemented with filtered olive oil (SDAO) was made to evaluate the possible isolation and growth of *Malassezia*.

The patient presented a slight clinical improvement until Day +14, when MALDI-TOF MS analysis was re-performed on the fungal culture and allowed us to identify the fungus as *M. furfur* (score 1.98). On the same day (Day +14), lactophenol blue mount made from a portion of the colony on CAN2 showed spherical to elliptical and teardrop-shaped yeast cells with short pseudohyphal elements and several monopolar budding cells (Fig. 1C). Daily doses of liposomal amphotericin (L-amB), at 3 mg/kg IV, were administered while the treatments with fluconazole and piperacillin/tazobactam were stopped due to negative blood cultures for *B. cereus* by BacT/Alert Virtuo® automated system.

Despite a clinical improvement, on Day +16 the patient presented hyperthermia, seizure and deterioration of the clinical condition. Empirical treatment with 1 g x 3 IV meropenem was started without improvement. On Day +18 after the hospitalization, the patient presented still neutropenic fever (neutrophils count 590/mmc), blood lactate dehydrogenase 3957 U/L, hypotension, dyspnea and tachypnea following by the exitus in the same day.

## 3 Discussion

*Malassezia* is the most prevalent fungal genus of the human skin microbiome [7]. Although *Malassezia* is a common commensal organism of the human skin flora, it is also known as a causative agent of cutaneous disorders such as dandruff and seborrheic dermatitis, as well as systemic infections particularly in immunocompromised individuals [8,9].

In recent years, the majority of invasive infections caused by the *Malassezia* genus have been linked to the lipid-dependent *M. furfur*. A comprehensive 12-month study on fungal bloodstream infections in critical care patients revealed a higher prevalence of *M. furfur* (2.1 %) compared to *Candida* spp. (1.4 %) in patients with complex underlying diseases or premature infants [10]. Unfortunately, due to inadequate diagnosis, invasive infections caused by *M. furfur* are likely to be underestimated.

Traditionally, *Malassezia* identification has relied on culture-based methods, by examining morphological and biochemical characteristics, i.e., the use of tweens and chromophore EL™, catalase activity, and growth at different temperatures [11]. However, these conventional microbiological approaches present some limitations. They struggle to differentiate between closely related species, are time-consuming, and have a high error rate. As a result, *Malassezia*-related disorders and infections are likely to be underdiagnosed in routine clinical laboratories. This is because standard non-lipid supplemented media, like Sabouraud glucose agar (SGA), do not support the growth of *Malassezia* and, therefore, delay the correct identification and treatment. Furthermore, the lack of species-level identification hampers our understanding of the epidemiology of *Malassezia*-related disorders and infections [[12], [13], [14]]. For these reasons, during the last five decades molecular based approaches and methods that identify the chemical imprint of the different species e.g., different Polymerase Chain Reaction (PCR) techniques, MALDI-TOF MS, as well as Raman spectroscopy have been applied to achieve fast and accurate fungal identification, including yeast fungi as *Malassezia* [15].

Despite that, the majority of the studies were focused on the cutaneous detection of *Malassezia*, while fast microbiology in systemic infections is negligible. In line with our case*,* other authors [16,17] have previously demonstrated that *M. furfur* fungemia can often be missed by the automated blood culture system BacT/Alert®. Particularly, out of 9 *M. furfur* fungemia cases reported by Iatta et al. [17], only 1 case of *M. furfur* fungemia was detected by the BacT/Alert® system. Also, Tetsuka et al. [18] have reported a case of *M. furfur* blood infection in a 3-year-old boy detected on blood-smear and not identified by automated blood culture systems. The low detention rate by automated systems could be due to the cytotoxic effect of human blood on yeast growth [18]. It has been demonstrated that the addition of 3 % palmitic acid in the hemoculture bottles might be able to overcome the inhibitory effect of both small (0.5 mL) and large (3 mL) volumes of blood, favoring *Malassezia* growth [19]. Finally, although molecular tools have been used to detect *Malassezia* yeasts from biological samples, no studies are reported in the literature on the molecular identification of *Malassezia* from direct blood specimens.

Thus, traditional direct microscopy and culture methods remain the gold standard for the detection of *M. furfur*. The lack of cutaneous tests to confirm the presence of *Malassezia* on the skin of our patient and the lack of antifungal susceptibility testing for *M. furfur* are some limitations of this study*.* Moreover, in this study the co-infection by *B. cereus* did not allow us to establish if the patient's death was attributable to bacteremia or fungemia or both. In conclusion, *M*. *furfu*r fungemia often remains undetermined by using automated blood culture systems. In our case, the direct microscopic examination of positive blood cultures, for the concomitant infection by *B. cereus,* helped us in diagnosing of systemic fungal infection that was confirmed as *Malassezia* bloodstream infection by traditional culture-based method and proteomic analysis by MALDI-TOF MS. Conversely, neither the automated blood culture system BacT/Alert Virtuo®, nor molecular methods were able to detect the presence of the fungus in the blood. Thus, there is unmet need for novel diagnostic tools to allow a rapid identification of this fungus from direct blood sample that is crucial for appropriate therapeutic interventions.

## Ethical form

The authors confirm that this material is original and has not been published in whole or in part elsewhere; that the manuscript is not currently being considered for publication in another journal; and that all authors have been personally and actively involved in substantive work leading to the manuscript and will hold themselves jointly and individually responsible for its content.

## CRediT authorship contribution statement

**Rosalba Petruccelli:** Writing – original draft, Software, Methodology, Conceptualization. **Terenzio Cosio:** Writing – original draft, Supervision, Software, Methodology, Conceptualization. **Valeria Camicia:** Investigation. **Carlotta Fiorilla:** Investigation. **Roberta Gaziano:** Writing – original draft, Supervision. **Cartesio D'Agostini:** Visualization, Supervision, Software.

## Declaration of competing interest

There are none.
